# Music-Induced Analgesia in Healthy Participants Is Associated With Expected Pain Levels but Not Opioid or Dopamine-Dependent Mechanisms

**DOI:** 10.3389/fpain.2022.734999

**Published:** 2022-04-04

**Authors:** Sigrid Juhl Lunde, Peter Vuust, Eduardo A. Garza-Villarreal, Irving Kirsch, Arne Møller, Lene Vase

**Affiliations:** ^1^Division for Psychology and Neuroscience, Department of Psychology and Behavioural Sciences, School of Business and Social Sciences, Aarhus University, Aarhus, Denmark; ^2^Center for Music in the Brain, Department of Clinical Medicine, Aarhus University, Aarhus, Denmark; ^3^The Royal Academy of Music Aarhus/Aalborg, Aarhus, Denmark; ^4^Laboratorio Nacional de Imagenología por Resonancia Magnética, Institute of Neurobiology, Universidad Nacional Autonoma de Mexico Campus Juriquilla, Queretaro, Mexico; ^5^Center of Functionally Integrative Neuroscience, Institute of Clinical Medicine, Aarhus University, Aarhus, Denmark; ^6^Program in Placebo Studies and Therapeutic Encounter, Beth Israel Deaconess Medical Center, Harvard Medical School, Boston, MA, United States; ^7^Department of Nuclear Medicine and PET Center, Institute of Clinical Medicine, Aarhus University and University Hospital, Aarhus, Denmark

**Keywords:** music-induced analgesia, endogenous opioids, dopamine, expectancy, context, placebo

## Abstract

Music interventions accommodate the profound need for non-pharmacological pain treatment. The analgesic effect of listening to music has been widely demonstrated across studies. Yet, the specific mechanisms of action have still to be elucidated. Although the endogenous opioid and dopamine systems have been suggested to play an important role, a direct link has not been established. In addition, the involvement of placebo mechanisms is likely while largely unexplored. We examined the analgesic effect of music in healthy participants (*n* = 48) using a 3 × 3 within-subjects design with pharmacological manipulations and a matched, auditory control for music. Participants were exposed to thermal pain stimuli while listening to three auditory excerpts: music (active condition), nature sound (matched, auditory contextual condition), and noise (neutral control condition). The participants rated their expected and perceived pain levels in relation to each of the auditory excerpts. To investigate the involvement of the endogenous opioid and dopamine systems, the test session was performed three times on separate days featuring a double-blind randomized oral administration of naltrexone (opioid antagonist), haloperidol (dopamine antagonist), and an inactive agent (control). Our results support an analgesic effect of music. Contrary to current hypotheses, neither of the antagonists attenuated the effect of music. Yet, the participants' expectations for pain relief predicted their perceived pain levels during the auditory excerpts—even when controlling for a gradual learning effect. In conclusion, we demonstrate that the analgesic effect of music is at least partially mediated by expectations of an analgesic effect—a core mechanism in placebo effects—but not by opioid and dopamine-dependent mechanisms.

**Clinical Trial Registration:**

www.clinicaltrials.gov, identifier: NCT03410563.

## Introduction

Facing a high prevalence of chronic pain worldwide and a rise in the use of pharmacological analgesics associated with profound human and societal costs (1–3), there is a great need for complementary, non-pharmacological pain treatments (4). Music can provide a safe and non-invasive intervention to reduce pain (5). The pain-relieving effect of music, termed music-induced analgesia (6), has been demonstrated in both acute (7–11) and chronic pain (12–15). Prevailing hypotheses regarding the mechanisms of action suggest that music may act to reduce pain through the release of endogenous opioids and dopamine (16–18). Yet, this has not been addressed directly by empirical investigations. In addition, due to methodological challenges, the general conclusion of music's eligibility in clinical practice may be at risk of overestimating the analgesic effect of music (19). Particularly, the lack of adequate control conditions may conceal a contribution from contextual treatment factors such as expectations about treatment efficacy (20, 21).

The assumption of neurotransmitter involvement in music-induced analgesia primarily derives from studies associating musical *pleasure* with endogenous opioid and dopamine transmission using ligand-based positron emission tomography and pharmacological agonist/antagonist paradigms (22–25). The opioid and dopamine systems contribute to a shared neurobiological foundation for pleasure and pain modulation (26), making them eligible candidates for mediating the analgesic effect of music. Among studies on music-induced analgesia, functional magnetic resonance imaging (fMRI) studies suggest that music taps into the descending modulation of pain (16, 18). Yet, although probable, these findings do not constitute direct evidence that this pain modulation is mediated by opioid and dopamine-dependent mechanisms. Moreover, the comparison between a music condition and a no-music condition (16, 18)—a standard design for examining the analgesic effect of music (27–29)—entails a risk of overestimating the specific effect of *music* itself (19).

In randomized controlled trials evaluating the effect of a pharmacological treatment, the active agent in question must show an effect beyond an inactive placebo (30). Put simply, this comparison against a placebo control allows for a distinction between improvement due to the specific treatment itself and improvement due to contextual factors—such as expectancy—embedded in the patient's perception of receiving the treatment (21, 30, 31). The importance of a contextual control is evidenced by findings demonstrating that expectations of treatment efficacy can double the analgesic effect of active pain medication (32). Among trials investigating non-pharmacological pain interventions such as surgery and acupuncture, the inclusion of matched contextual conditions omitting the treatment specific characteristics is currently being debated and implemented (33–39), and the general need for well-controlled trials in relation to alternative or complementary pain interventions is being recognized (40). As expressed in a recent article on grand challenges in non-pharmacological treatment of pain, it is essential to both demonstrate an effect of these interventions beyond a placebo effect, and to specify their biological underpinnings (40). At this point, however, only few studies have used a contextual control or taken expectations for pain relief into account when evaluating the analgesic effect of music (8, 41–43). Thus, it is largely unknown to which extent placebo mechanisms contribute to this effect.

**Figure 1:**
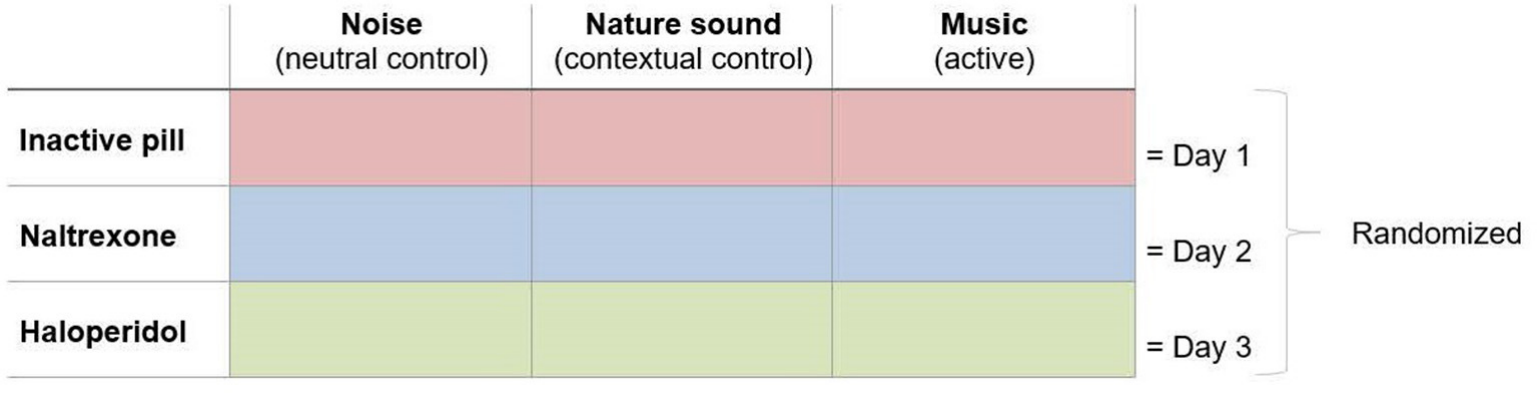
Study design. On each test day, participants were exposed to individually calibrated pain stimuli while listening to noise, nature sound, and music in randomized order accompanied by administration of either an inactive agent, naltrexone, or haloperidol in a double-blind, randomized order.

The present study was undertaken to investigate the role of neurotransmitter activity and expectancy in music-induced analgesia in healthy participants exposed to thermal stimuli. Using a 3 × 3 within-subjects design (Figure 1), each participant rated their expected and perceived pain levels in relation to 3 auditory excerpts: music (active condition), nature sound (matched, auditory contextual condition), and noise (neutral control condition). This was repeated on 3 separate days to test the involvement of the endogenous opioid and dopamine systems pharmacologically by double-blind administration of naltrexone (opioid antagonist), haloperidol (dopamine antagonist), and an inactive agent (control). Order of both auditory and pharmacological conditions was randomized and counterbalanced. It was hypothesized that the analgesic effect of music would be attenuated by naltrexone and haloperidol, respectively—i.e., suggesting that opioids and dopamine mediated the effect—and that expectations for pain relief would contribute to the magnitude of the analgesic effects observed across auditory excerpts.

## Materials and Methods

### Participants

Forty-eight healthy participants (21 males, 27 females) aged 19–56 years (mean = 24.65, SD = 7) completed the study (see Supplementary Materials and Methods for dropouts). A power calculation based on a previous study by Villarreal et al. (42) showed that 50 participants would be sufficient, α (two-tailed) = 0.05, β = 0.80. Due to the randomization and counterbalanced distribution of conditions (Supplementary Figure 1), however, 48 participants were included in the study.

Eligibility was assessed using the following inclusion criteria: Normal health, normal hearing, age 18–60 years, and fluent in Danish. Exclusion criteria were chronic pain, other medical, neurological, or psychiatric conditions, use of antidepressants, daily use of analgesic medication or 24 h prior to testing, substance abuse, pregnancy, and enrollment in/completion of an education in musicology/at a music academy. In average, participants reported 4.56 years of musical experience (SD = 5.39) and scored 74.29 (SD = 9.55) on the Musical Ear Test (44) measuring their musical competence (score range: 0–100). Smoking was not included as an exclusion criterion. However, participants were not allowed to smoke during their participation in the study.

Prior to inclusion in the study, participants were informed (verbally and in writing) that the aim of the study was to investigate the analgesic effect of music and nature sound. Specifically, the participants were told that both of these auditory excerpts had been associated with pain relief in a previous study (42). Noise was introduced as a neutral control condition (see Supplementary Materials and Methods for scripted information). Participants were also informed that we wanted to examine whether the medications (naltrexone and haloperidol) would modify their pain experience. However, they were blinded to the hypotheses regarding antagonism and a matched auditory condition to control for contextual factors.

Participants gave informed consent before entering any study procedures and received monetary compensation (200 DKK/test day; 600 DKK in total). The study was approved by The Central Denmark Region Committees on Health Research Ethics (1–10–72–317–16) and registered at ClinicalTrials.gov (Identifier: NCT03410563).

### Randomization

Using random draw, participants were assigned to different groupings specifying a counterbalanced distribution of conditions across test days (Latin and Graeco-Latin squares; see detailed information in the Supplementary Materials and Methods and in Supplementary Figure 1). The distribution of pharmacological manipulations was blind for everyone involved in the study until completion of the data analysis—except for 2 consulting physicians who broke the blinding code only in case a participant felt unwell during testing. Aside from these consultations, the physicians did not have contact with the participants and were not involved in the data analysis.

### Procedures

#### Thermal Stimuli

Participants were exposed to painful thermal stimuli produced by a 3 × 3 contact thermode (Pathway Model ATS; Medoc Ltd. Advanced Medical System, Israel) placed on the anterior surface of the forearm. Calibration trails were performed to obtain individual pain stimuli reflecting a perceived pain intensity of 60–70 mm (moderate to high pain) on a 0–100 mm mechanical visual analogue scale (42, 45) (see Supplementary Materials and Methods for detailed information). The individually calibrated temperature was kept constant for each participant in all test sessions. Each auditory excerpt was accompanied by 3 thermal stimuli consisting of a 16-s plateau with a rise and fall time of 2°C/s and a baseline temperature of 35°C during rest intervals (42) (Supplementary Figure 2).

#### Auditory Excerpts

Three auditory excerpts were employed in different order on all 3 test days. The active music condition consisted of a Mozart string composition, the matched, auditory contextual condition consisted of the sound of water, and the control condition consisted of pink noise. Pink noise was included as a neutral auditory input, whereas the music piece and the nature sound were chosen for their compatibility on 3 emotional measures (valence, liking, and arousal) obtained in a previous study (42). Aside from this compatibility, one important element set the two conditions apart. When we listen to music—contrary to random sound—the intentional compositions of, e.g., harmonies, melodies, and rhythms cause us to build expectations for what will come next (46, 47). Musical pleasure can come from the confirmation or skillful violation of these expectancies (48). This element of musical expectancy is considered to be a key factor in the musical experience (49), and the anticipation of peak pleasure moments during music listening has been associated with dopamine release (24). By administering a nature sound without musical structure that enable anticipation, nature sound was conceptualized as a matched, auditory contextual control for music. Thus, the nature sound and the music piece shared the fundamental transmission of content (constituting a pleasant auditory stimulus) without sharing the actual content and element of musical expectancy (see Supplementary Materials and Methods for detailed information).

Each auditory excerpt was peak normalized and lasted 300 s (42). The 3 thermal stimuli were delivered during the last 150 s (Supplementary Figure 2).

#### Pharmacological Manipulations

Three identical white capsules containing an inactive agent, naltrexone (25 mg), or haloperidol (3 mg) were administered orally with a glass of water (200 ml) 2 h prior to testing to allow the medications to take effect (50, 51). All test sessions were arranged to take place at approximately the same time for each participant across the 3 test days (mean divergence in min = 56.88; SD = 41.62), and the test days were placed minimum 3 days apart in order for the medication to wear off (see Supplementary Materials and Methods for detailed information and Supplementary Table 1 for reports of adverse events).

### Measures

#### Ratings of Expected and Perceived Pain Intensity and Pain Unpleasantness

In order to examine the participants' expectations as a predictor of the analgesic effects, participants were asked to rate their expected pain intensity and pain unpleasantness immediately before the administration of each auditory excerpt knowing what they were about to listen to. Expectancy ratings were obtained on mechanical visual analogue scales (M-VAS; 0–100 mm) anchored by the descriptors “no pain”/”no unpleasantness” (=0) and “worst imaginable pain”/“worst imaginable unpleasantness” (=100) (52, 53). After each thermal stimulus, participants were asked to rate their perceived pain intensity and pain unpleasantness on the M-VAS (52) (Supplementary Figure 2).

#### Emotional Measures

To test the compatibility in emotional ratings between music and nature sound, participants were asked to rate all auditory excerpts on an 11-point Likert scale for valence (0 = unpleasant, 10 = pleasant), liking (0 = do not like, 10 = like), and arousal (0 = relaxing, 10 = stimulating) on all 3 test days immediately after listening to each of the excerpts (6, 42).

### Statistical Analysis

We assumed a normal distribution of data based on the Kolmogorov–Smirnov test. Two-way repeated measures ANOVAs and pairwise comparisons were conducted to determine the differences in pain ratings (for pain intensity and pain unpleasantness, respectively) across auditory excerpts and pharmacological manipulations. Furthermore, two-way repeated measures ANOVAs and pairwise comparisons were conducted to determine the differences in expectancy (for expected pain intensity and expected pain unpleasantness, respectively) across auditory excerpts and pharmacological manipulations. Pearson correlation analyses were conducted to determine the association between pain ratings and pain expectancy in relation to the first auditory excerpt on test day 1 (regardless of type of auditory input and regardless of pharmacological manipulations) in order to examine this association without preceding familiarity with the test situation. To examine this association on test days 2 and 3, respectively, zero-order correlation analyses were conducted to examine how pain levels were associated with prior pain experience and pain expectancy. Furthermore, controlled partial correlation analyses were conducted to examine the association between pain levels and pain expectancy on test days 2 and 3, respectively, when controlling for prior pain experience. In order to examine how expectancy and prior pain experience predicted later expectancy and pain ratings across the 3 test days, path regression analyses were conducted for each of the 3 auditory excerpts (for pain intensity and pain unpleasantness, respectively).

Secondary, two-way repeated measures ANOVAs and pairwise comparisons were conducted to determine the differences in emotional ratings (valence, liking, and arousal, respectively) across auditory excerpts and pharmacological manipulations, and Pearson correlation analyses examined the association between the emotional ratings and pain levels (pain intensity and pain unpleasantness, respectively) during each of the auditory excerpts.

## Results

### Perceived Pain Intensity and Unpleasantness

**Figure 2:**
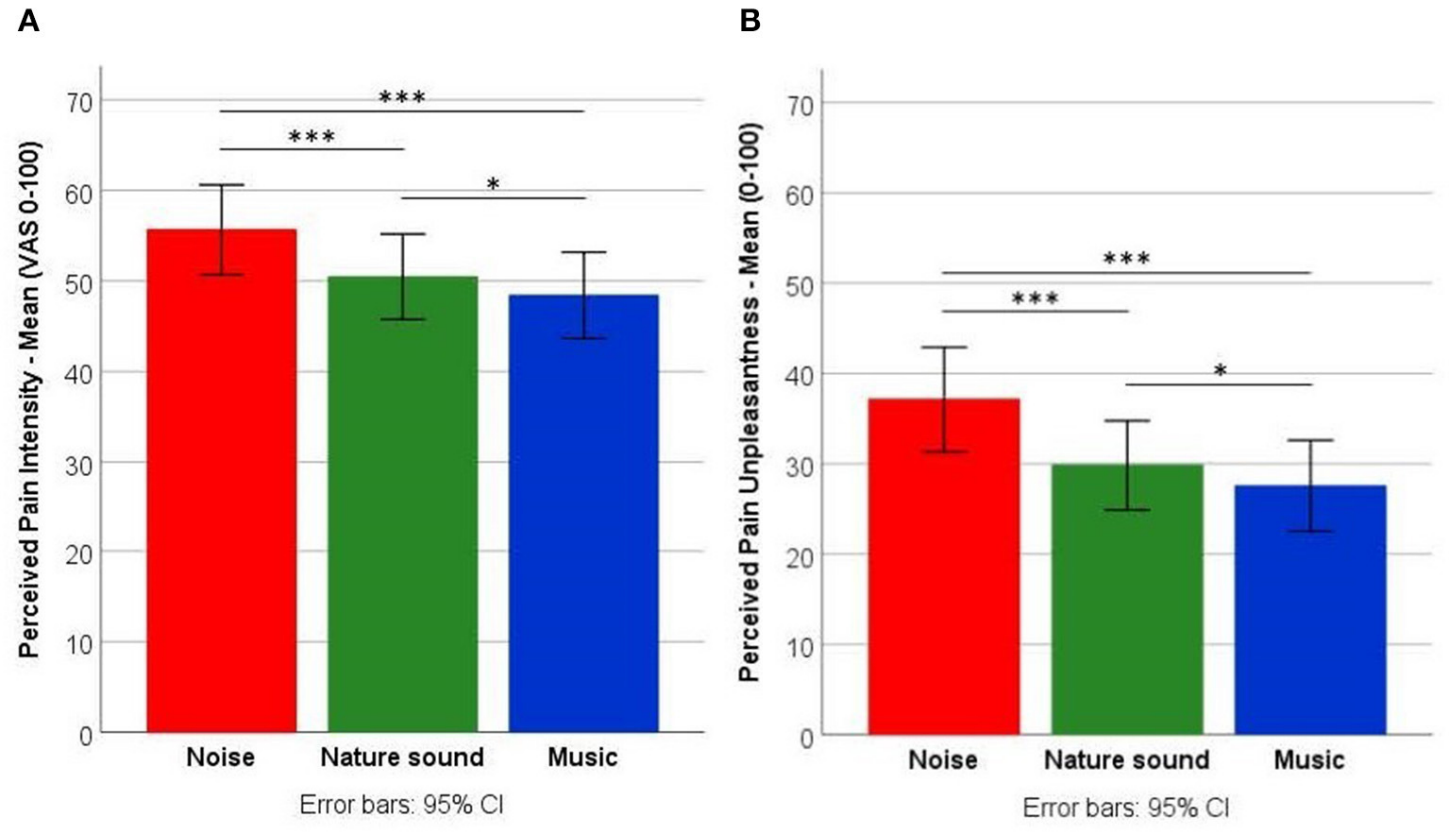
Pain levels. Comparisons of noise, nature sound, and music on **(A)** pain intensity and **(B)** pain unpleasantness (regardless of pharmacological manipulations). *p < 0.05; ***p < 0.001.

Results of the two-way repeated measures ANOVA for perceived pain showed significant main effects for the type of auditory excerpt in relation to pain intensity [*F*_(2, 94)_ = 28.96, *p* < *0*.001, eta^2^ = 0.381], and pain unpleasantness [*F*_(1.55, 72.65)_ = 32.52, *p* < *0*.001, eta^2^ = 0.409, using the Greenhouse-Geisser correction]. Bonferroni-corrected contrasts revealed that music and nature sound reduced pain intensity (*p* < *0*.001) and pain unpleasantness (*p* < *0*.001) significantly compared with noise. Ratings of pain intensity (*p* = 0.046) and pain unpleasantness (*p* = 0.04) were significantly lower when participants listened to music than when they listened to nature sound (Figure 2). There were no significant main effects of pharmacological manipulations [pain intensity: *F*_(2, 94)_ = 0.14, *p* = 0.869, eta^2^ = 0.003; pain unpleasantness: *F*_(1.68, 79.12)_ = 0.053, *p* = 0.92, eta^2^ = 0.001, using the Greenhouse-Geisser correction], and there were no significant interactions between the type of auditory excerpt and the pharmacological manipulations [pain intensity: *F*_(4, 188)_ = 0.14, *p* = 0.968, eta^2^ = 0.003; pain unpleasantness: *F*_(4, 188)_ = 0.73, *p* = 0.570, eta^2^ = 0.015]. See Supplementary Figure 3 and Supplementary Table 2 (mean scores).

### Expected Pain Intensity and Unpleasantness

**Figure 3:**
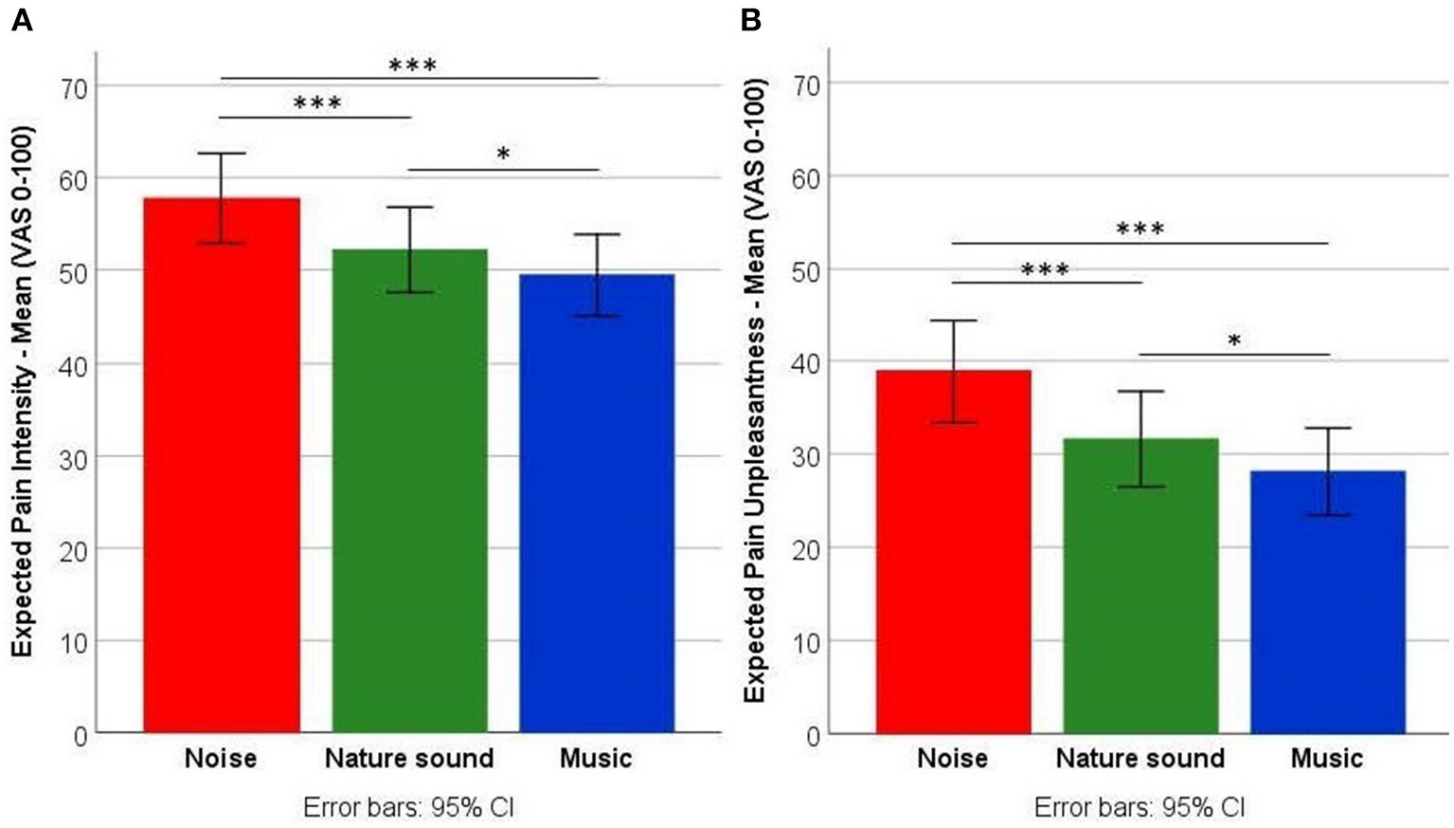
Expected pain levels. Comparisons of noise, nature sound, and music on **(A)** expected pain intensity and **(B)** expected pain unpleasantness (regardless of pharmacological manipulations). *p < 0.05; ***p < 0.001.

Results of the two-way repeated measures ANOVA for expected pain showed significant main effects for the type of auditory excerpt in relation to pain intensity [*F*_(2, 94)_ = 36.78, *p* < *0*.001, eta^2^ = 0.439] and pain unpleasantness [*F*_(2, 94)_ = 36.33, *p* < *0*.001, eta^2^ = 0.436]. Bonferroni-corrected contrasts revealed that participants expected significantly lower pain intensity (*p* < *0*.001) and pain unpleasantness (*p* < *0*.001) from music and nature sound compared to noise. Also, the participants expected significantly lower pain intensity (*p* = 0.026) and pain unpleasantness (*p* = 0.011) from music compared to nature sound (Figure 3). There were no significant main effects of pharmacological manipulations [pain intensity: *F*_(2, 94)_ = 0.24, *p* = 0.787, eta^2^ = 0.005; pain unpleasantness: *F*_(2, 94)_ = 0.07, *p* = 0.929, eta^2^ = 0.002], and there was no significant interaction between the type of auditory excerpt and the pharmacological manipulations [pain intensity: *F*_(3.25, 152.63)_ = 1.60, *p* = 0.189, eta^2^ = 0.033, using the Greenhouse-Geisser correction; Pain unpleasantness: *F*_(3.21, 150.94)_ = 1.28, *p* = 0.283, eta^2^ = 0.027, using the Greenhouse-Geisser correction]. See Supplementary Figure 4 and Supplementary Table 3 (mean scores).

### Expected and Perceived Pain Intensity and Unpleasantness on Test Day 1

Given the non-significant effect of the pharmacological manipulations, we tested the relationship between expected and perceived pain intensity and unpleasantness by day, examining the first auditory excerpt presented to the participants, to explore the relationship between expectations and perception of pain without interference of previous experience from taking part in the study. Results of Pearson correlation analyses for the first auditory excerpt on test day 1 (regardless of the type of auditory excerpt and pharmacological manipulations) showed that expected pain intensity and perceived pain intensity were strongly correlated, *r*_(46)_ = 0.66, *p* < *0*.001, and that expected pain unpleasantness and perceived pain unpleasantness were strongly correlated, *r*_(46)_ = 0.83, *p* < *0*.001. See Supplementary Figure 5.

### Distinguishing Expectancy From Prior Pain Experience on Test Day Two and Three

**Figure 4:**
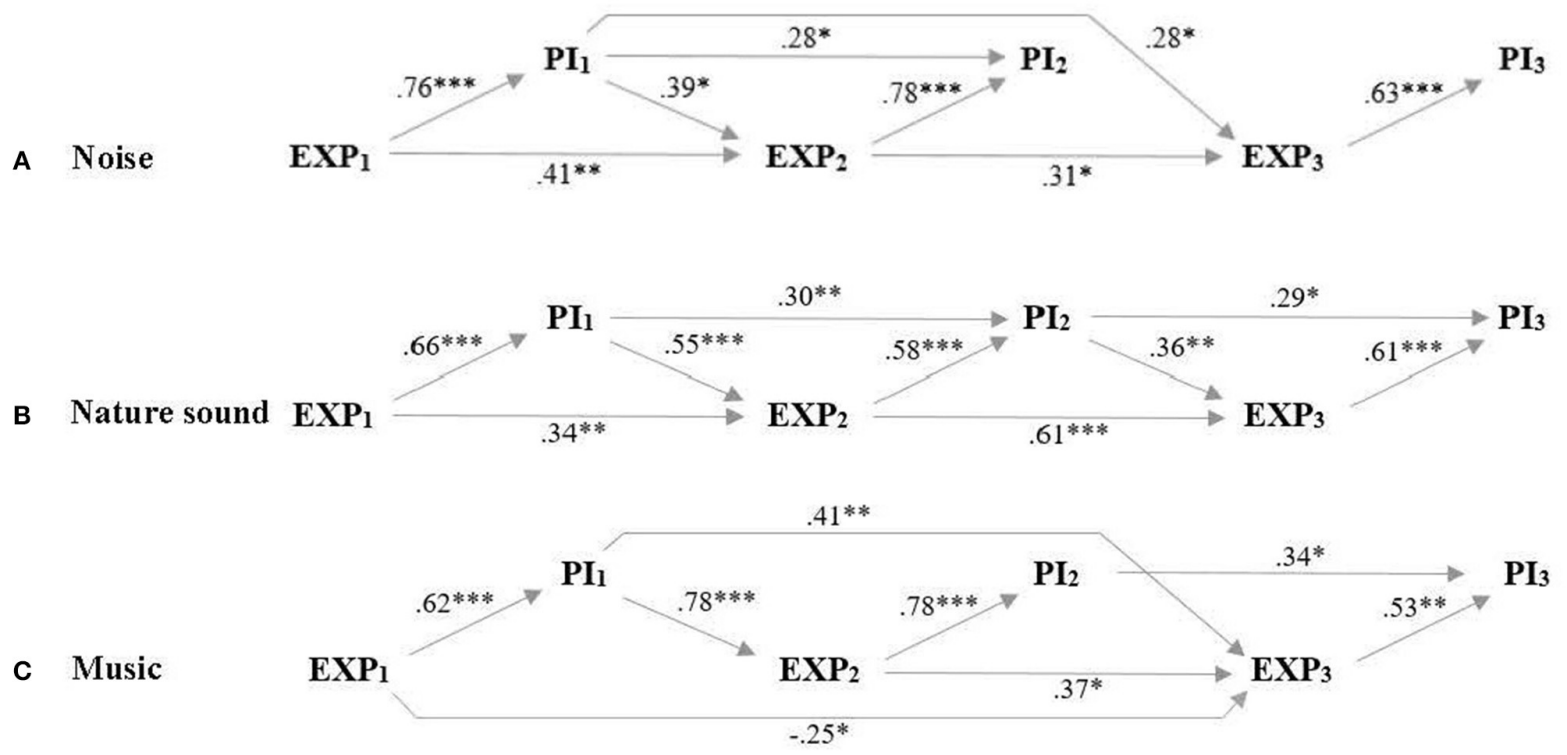
Perceived and expected pain intensity. Path regression analysis of expectancy ratings on the 3 test days (EXP1, EXP2, EXP3) and perceived pain intensity on the 3 test days (PI1, PI2, PI3) as predictors for later expectancy and pain ratings when participants listened to **(A)** noise, **(B)** nature sound, and **(C)** music. Arrows and beta-values mark significant predictions and demonstrate that expected pain intensity predicted perceived pain intensity on all respective test days when including all previous expectancy and pain intensity ratings in the regression model. *p < 0.05; **p < 0.01; ***p < 0.001.

**Figure 5:**
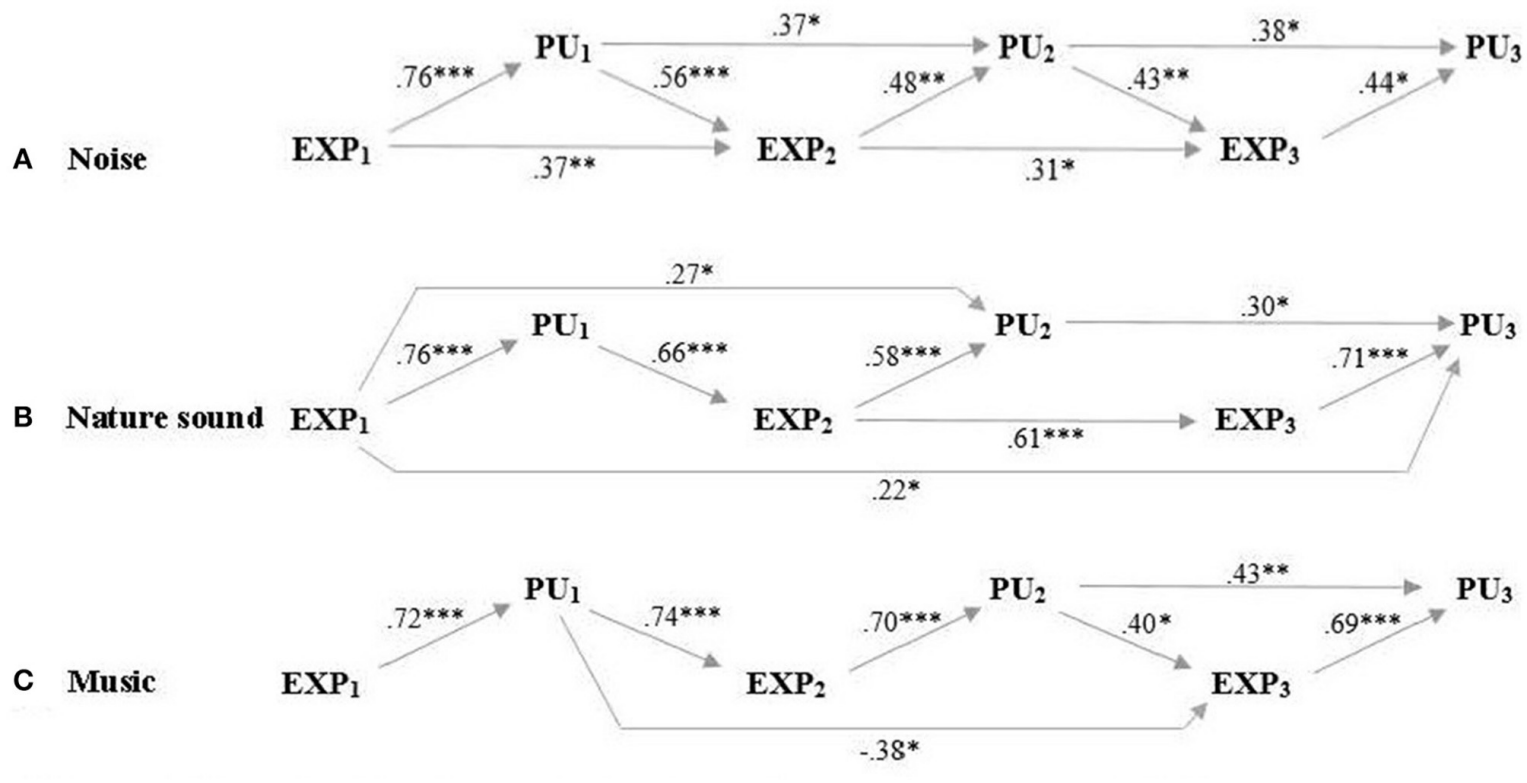
Perceived and expected pain unpleasantness across test days. Path regression analysis of expectancy ratings on the 3 test days (EXP_1_, EXP_2_, EXP_3_) and perceived pain unpleasantness on the 3 test days (PU_1_, PU_2_, PU_3_) as predictors for later expectancy and pain ratings when participants listened to **(A)** noise, **(B)** nature sound and **(C)** music. Arrows and beta-values mark significant predictions and demonstrate that expected pain unpleasantness predicted perceived pain unpleasantness on all respective test days when including all previous expectancy and pain unpleasantness ratings in the regression model. *p < 0.05; **p < 0.01; ***p < 0.001.

Given the 3 × 3 within-subjects study design in which the participants were tested on 3 separate test days, we tested how perceived pain intensity and unpleasantness (on test day 2 and 3, respectively) were associated with prior pain experience (perceived pain intensity or unpleasantness on the previous test day) and pain expectancy (expectations for pain intensity or unpleasantness on the present test day). Results of zero-order correlations showed that both prior pain experience and expectations were strongly correlated with perceived pain intensity and unpleasantness (Tables 1, 2). Results of controlled partial correlations, controlling for prior pain experience and pain expectancy, respectively, showed that expectations for pain intensity and unpleasantness were still strongly correlated with perceived pain intensity (Table 1) and unpleasantness (Table 2) when controlling for prior pain experience. Results of path regression analyses, examining how expectancy and prior pain experience predicted later expectancy and pain ratings, showed that expectations for pain intensity (Figure 4) and unpleasantness (Figure 5) on the present test day significantly predicted perceived pain intensity and unpleasantness when including all previous expectancy and pain ratings in the regression model.

**Table 1:** Correlations between expected and perceived pain intensity and between prior and perceived pain intensity across auditory excerpts.

			**PI**_**2**_		**PI**_**3**_	
			**Zero**	**Partial**	**Zero**	**Partial**
Noise	PI_prior_	*r*	0.73^***^	0.30^*^	0.76^***^	0.15
	EXP	*r*	0.88^***^	0.75^***^	0.89^***^	0.72^***^
Nature sound	PI_prior_	*r*	0.81^***^	0.42^**^	0.90^***^	0.43^**^
	EXP	*r*	0.87^***^	0.67^***^	0.94^***^	0.69^***^
Music	PI_prior_	*r*	0.74^***^	0.19	0.83^***^	0.47^**^
	EXP	*r*	0.89^***^	0.74^***^	0.86^***^	0.57^***^

*Zero-order correlations (Zero) and controlled correlations (Partial) between expected pain intensity (EXP) and perceived pain intensity on test days 2 and 3 (PI_2_ and PI_3_) and between prior pain intensity (PI_prior_) and perceived pain intensity on test days 2 and 3 (PI_2_ and PI_3_) for noise, nature sound, and music*.

* *p < 0.05*;

** *p < 0.01*;

*** *p < 0.001*.

**Table 2:** Correlations between expected and perceived pain unpleasantness and between prior and perceived pain unpleasantness across auditory excerpts.

			**PU**_**2**_		**PU**_**3**_	
			**Zero**	**Partial**	**Zero**	**Partial**
Noise	PU_prior_	*r*	0.81^***^	0.38^**^	0.87^***^	0.40^**^
	EXP	*r*	0.83^***^	0.47^**^	0.88^***^	0.50^***^
Nature sound	PU_prior_	*r*	0.80^***^	0.39^**^	0.85^***^	0.41^**^
	EXP	*r*	0.87^***^	0.64^***^	0.90^***^	0.66^***^
Music	PU_prior_	*r*	0.68^***^	0.28	0.83^***^	0.43^**^
	EXP	*r*	0.84^***^	0.70^***^	0.90^***^	0.71^***^

*Zero-order correlations (Zero) and controlled correlations (Partial) between expected pain unpleasantness (EXP) and perceived pain unpleasantness on test days 2 and 3 (PU_2_ and PU_3_) and between prior pain unpleasantness (PU_prior_) and perceived pain unpleasantness on test days 2 and 3 (PU_2_ and PU_3_) for noise, nature sound, and music*.

** *p < 0.01*;

*** *p < 0.001*.

### Emotional Measures

Music and nature sound were compatible (non-significant differences in ratings) on valence and liking, whereas nature sound was rated to be significantly more relaxing (low arousal) than music. Both music and nature sound were rated significantly higher on valence and liking and significantly lower on arousal compared with noise. See **Supplementary Results** for results of the analyses, Supplementary Table 4 for mean scores, and Supplementary Table 5 for correlations between emotional ratings and pain ratings.

## Discussion

Our results suggest that music relieves pain regardless of opioid and dopamine-dependent mechanisms. Importantly, the analgesic effect of music was strongly predicted by the participants' expectations for pain relief, pointing to a substantial contribution from contextual factors (21) not associated with music *per se*. These results encourage a new understanding of the mechanisms that drive music-induced analgesia and emphasize the importance of adequate control conditions when evaluating the analgesic effect of music.

Overall, the findings of the present study substantiate an analgesic effect of music as shown in previous studies (6, 8, 11, 16, 41, 42). Participants reported significantly lower pain levels when listening to music than when listening to nature sound and noise. However, pertaining to the underlying neurobiological mechanisms—and contrary to our first hypothesis—neither of the antagonists attenuated this analgesic effect of music. In one fMRI study on music-induced analgesia (16), playing pleasant and preferred music to healthy participants exposed to experimental pain was associated with a decrease in subjective pain ratings as well as an increase in blood oxygen level dependent (BOLD) responses in anatomic proximity to the periaqueductal gray (PAG). Considering the central role of the PAG in the descending pain modulatory system (54) together with its high expression of endogenous opioids and opioid receptors (55), these findings are compatible with the hypothesis that music activates descending pain modulation through the release of endogenous opioids (16, 18). Furthermore, in another fMRI study, pleasant and preferred music was found to activate the nucleus accumbens (NAc) and alter connectivity between NAc and key regions in the corticostriatal circuits during pain onset (56). When comparing these findings to studies that associate dopamine release in NAc with music-induced pleasure (24) and substantiate the role of dopamine signaling in pain (57), it seems likely that dopamine is involved in music-induced analgesia. Importantly, however, the fMRI BOLD response may be interpreted as a proxy for neural activity but with no specification of *neurotransmitter* activity (25, 58), leaving no direct evidence to suggest that music in fact activates the descending pain modulatory system through the release of endogenous opioids and dopamine. Thus, although interpretations in favor of an opioid and dopamine mediated analgesic effect of music seem highly probable based on indirect measures, our pharmacological paradigm—targeting neurotransmitter activity *directly*—challenges this interpretation and encourages more investigations to specify the role of neurotransmitters.

Adding to the methodological considerations, future studies may also benefit from specifying the contribution from contextual factors when evaluating the analgesic effect of music. Our findings suggest that a considerable part of this effect may not be ascribable to the music excerpt, but rather to the participants' expectations. In agreement with our second hypothesis, our results show consistently strong associations between expected and perceived pain intensity and unpleasantness (Tables 1, 2) for all 3 auditory excerpts throughout the study. These associations were also significant when controlling for prior pain experiences from previous test days arguing that the pain-relieving effects observed throughout the study were not attributable to a gradual learning effect (i.e., an effect of prior pain levels). Furthermore, the path regression analyses (Figures 4, 5) establish expectancy as a significant predictor of perceived pain intensity and unpleasantness for all 3 auditory excerpts on each of the 3 test days while, at the same time, demonstrating a continued interplay between expectancy and experience carrying over to subsequent pain expectations. For further discussion of the contribution from contextual factors in music-induced analgesia, see (19).

Together, these findings accentuate the importance of not only demonstrating an effect of music, but also specifying the factors contributing to the effect. Although participants *experienced* significantly lower pain levels during music compared with nature sound and noise, they also *expected* significantly lower pain levels in relation to music compared with nature sound and noise (as demonstrated by similar patterns in Figures 2, 3). Thus, adding to mixed findings from previous studies (8, 41, 43), results from this study suggest that expectations of pain relief—a core element in placebo effects (59)—contribute significantly to the analgesic effect of music.

### Limitations and Implication for Future Research

When discussing the current results, some methodological limitations and implications may be addressed. Firstly, a possible dose-dependent effect should be considered in relation to results on opioid and dopamine-dependent mechanisms. Whereas the 3 mg haloperidol used in the study corresponds to the recommendations for single doses in healthy participants (50, 60), the 25 mg naltrexone balances dose efficacy and risk of adverse events. In a study examining the role of endogenous opioids in music and emotion, Mallik and colleagues argued for 50 mg naltrexone as lowest effective dose (22). Importantly, however, in our pilot study, 50 mg caused substantial discomfort and adverse events among participants, and even the 25 mg naltrexone administered in this main study was associated with adverse events (Supplementary Table 1) substantiating that the antagonist did take effect. Adding to these considerations, Lee and colleagues (51) suggested that a dose of 50 mg oral naltrexone may be far greater than what is needed to occupy opiate receptors and that lower doses may be sufficient and result in fewer side effects. Accordingly, on the one hand, the dose of naltrexone necessitates some caution when interpreting the results of the present study in regard to opioid-dependent mechanisms. On the other hand, it cannot be ruled out that the experience of adverse events following haloperidol and naltrexone may have had an effect on the participants' overall experiences (e.g., expected and perceived effects) on the present and following test days. Furthermore, despite results showing no effects of the pharmacological manipulations (i.e., no attenuation of analgesic effects), it should be noted that no physiological criteria were used to assess that the action of the medication had actually ceased during the washout periods (between test days).

Secondly, the implementation of carefully matched, auditory contextual controls for music composes a new area of research within studies on music-induced analgesia, and various modifications may be pursued in future study designs. Exemplifying this, it would be beneficial to include measures of baseline pain levels without auditory stimuli (silence). This would also allow us to verify if pink noise indeed acts as a neutral control with no positive or negative effect on pain levels—compatible to previous findings showing no differences in pain levels when comparing white noise to silence (61). Other approaches to specifying the role of specific and contextual factors may be to vary and directly compare the outcomes of different music parameters and characteristics (62), to vary the information given about the different auditory excerpts (e.g., a mixed design in which only some participants receive information on the hypothesized analgesic effects of music and nature sound) (63–65) and explicitly targeting other contextual and emotional factors such as familiarity and preference (8, 19).

The auditory paradigm used in this study (i.e., the specific auditory excerpts with an exposure phase of 5 min) is based on a previous study showing an analgesic effect of music and nature sound compared to pink noise (42). It should be recognized, however, that there is generally no consensus across the literature as to how long these exposures should be—ranging from, e.g., 4 min in experimental studies with healthy participants (8) to 15–60 min in clinical studies on patients with chronic pain (66–68). Furthermore, whereas previous studies investigating neural underpinnings of music-induced analgesia and musical pleasure have used participants' favorite music (16, 56), participants in this study all listened to the same auditory excerpts. This inclusion of researcher-chosen music may be regarded as both a disadvantage and advantage. On the one hand, self-chosen music has been suggested to be superior to researcher-chosen music in relieving pain (12). On the other hand, researcher-chosen music may be more compatible with clinical applications of music requiring no further preparation. Moreover, although our data on pharmacological antagonism and neurotransmitter-dependent mechanisms in music-induced analgesia should be interpreted in relation to researcher-chosen music, the pharmacological paradigm used in the study can be applied also in relation to highly preferred and familiar music.

Finally, acknowledging that findings obtained in healthy participants exposed to acute pain may not necessarily be transferred to patients experiencing chronic pain (69, 70), more studies are needed to specify similarities and dissimilarities in the mechanisms underlying music-induced analgesia in acute and chronic pain.

Independently of the type of music or study population, however, future study designs should take into account that a substantial part of the analgesic effect may be explained by contextual factors that exceed the characteristics and qualities of music. Thus, in order to fully evaluate the beneficial effects of music *per se*, the inclusion of carefully matched, auditory contextual controls may be utilized further to elaborate on how music acts to relieve pain.

## Conclusion

In conclusion, the present findings show that expectations for pain relief is an important predictor for the analgesic effect of music—as well as for other auditory material. They also suggest that the assumed key role of the endogenous opioid and dopamine systems in music-induced analgesia has to be tested directly in more studies before we can infer if and how they contribute to this analgesic effect. The methodological approach used in this study provides a model for further investigations of music-induced analgesia, the mechanisms by which music acts to relieve pain as well as the specific—and contextual—factors contributing to this effect.

## Data Availability Statement

The raw data supporting the conclusions of this article will be made available by the authors upon request, without undue reservation.

## Ethics Statement

The study was reviewed and approved by the Central Denmark Region Committees on Health Research Ethics. The participants provided their written informed consent to participate in the study.

## Author Contributions

SJL: data acquisition, analysis, and drafting the article. SJL, PV, EAG-V, and LV: conception and design of the study. PV and EAG-V: revising the article critically for important intellectual content. IK and LV: data analysis. IK, LV, and SJL: interpretation. IK and AM: revising the article critically for important intellectual content. AM: medical supervision and responsibility during data acquisition. LV: drafting the article. All authors contributed to the article and approved the submitted and final version.

## Funding

Center for Music in the Brain is funded by the Danish National Research Foundation (DNRF117).

## Conflict of Interest

The authors declare that the research was conducted in the absence of any commercial or financial relationships that could be construed as a potential conflict of interest.

## Publisher's Note

All claims expressed in this article are solely those of the authors and do not necessarily represent those of their affiliated organizations, or those of the publisher, the editors and the reviewers. Any product that may be evaluated in this article, or claim that may be made by its manufacturer, is not guaranteed or endorsed by the publisher.
