# Supplementary material for: Music-Induced Analgesia in Healthy Participants Is Associated With Expected Pain Levels but Not Opioid or Dopamine-Dependent Mechanisms
Source: Front Pain Res (Lausanne). 2022 Apr 4;3:734999. doi: 10.3389/fpain.2022.734999 (PMC9013883; doi:10.3389/fpain.2022.734999)
Supplement: Supplementary file 1 [file Data_Sheet_1.pdf]

Supplementary Material

1 Supplementary Materials and Methods

1.1 Participants and Randomization

From the initial recruitment, 1 participant dropped out after test day 1 (due to acute disease), and 1 participant dropped out after test day 2 (due to busyness). Thus, to obtain a counterbalanced design with 48 participants, 2 additional participants were enrolled in the study to complete all 3 test days. All 48 participants were included in all analyses.

Participants were recruited through a research recruitment website and through advertisements on campus (Aarhus University) and in a local newspaper from January, 2018 to March, 2018. Sigrid Juhl Lunde enrolled all participants in the study. The random allocation sequence was done by a biostatistician (not part of the project group), and the participants were assigned to the different sequences of conditions using random draw (and ID numbers only) managed by student assistant Lærke Søe Christensen.

1.2 Thermal Stimuli

In order to control for individual differences in pain processing and perception, calibration trails were performed before the actual testing. The calibration consisted of successive pain stimuli on the right forearm with a gradual increase of 1 °C starting at 42 °C with a maximum of 48.5 °C (1). Each thermal stimulus lasted 16 seconds with intervals of 30 seconds rest in between stimuli. After each stimulus, participants rated their pain intensity and pain unpleasantness on mechanical visual analogue scales (M-VAS; 0-100 mm). On this basis, an individually calibrated temperature was computed for each participant reflecting a perceived pain intensity of 60-70 mm (moderate to high pain) on the M-VAS (2, 3). During test sessions, the thermode was placed on the left forearm with slight changes in position after every 3rd thermal stimulus (between auditory excerpts) in order to avoid habituation.

1.3 Auditory Excerpts

In agreement with a previous study by Villarreal et al. (2), the active music condition consisted of a Mozart string composition (*"String Quartet No. 1 in G major, K 80/73f (1770) – Adagio"*), the matched, auditory contextual condition consisted of the sound of water (Sound effects Library, Sound Ideas https://www.sound-ideas.com), and the neutral control condition consisted of pink noise. The nature sound in the matched, auditory contextual condition provided an auditory input without any musical components such as tempo, mode, structure, and development through harmony, melody, and rhythm, causing us to build expectations for what will come next (4).

The music piece and the nature sound were matched based on their compatibility on 3 emotional measures (valence, liking, and arousal). Specifically, in the previous study and pilot study by

Villarreal et al. (2), participants' ratings of the Mozart composition and the sound of water reflected equally high valence and liking ratings and low arousal.

The auditory excerpts were introduced to the participants using the same scripted information to ensure stringency. On their first test day, the participants were told that "you will now listen to [the musical piece/the nature sound] that has previously been shown to reduce pain significantly in some participants" (5, 6). Pink noise was introduced as a control condition by telling the participants that "you will now listen to a noise clip that acts as a control condition for music and nature sound". On their second and third test day, participants were told that "you will now listen to same [musical piece/ nature sound/control condition] that you listened to on your previous test day".

1.4 Pharmacological Manipulations

The inactive agent, naltrexone, and haloperidol (from *Glostrup Pharmacy*, Denmark) were delivered in identical white capsules to ensure double-blind administration. The capsules were administered from identical pill boxes stating ID number and test day (1, 2, and 3 with a capsule for each day). Participants were informed that they would receive both the inactive agent and each of the active medications during their participation in the study (one per test day), without knowing the order of administration. Only the 2 consulting physicians were able to break the blinding code in case a participant felt unwell during testing in order for the physicians to check whether this was linked with the administration of naltrexone/haloperidol (see Supplementary Table 1 for adverse events).

All testing took place at the Center of Functionally Integrative Neuroscience at Aarhus University Hospital, Denmark, from January, 2018 to March, 2018, and 1 of the physicians was always available during testing.

2 Supplementary Results

2.1 Perceived and Expected Pain Intensity and Unpleasantness

The Greenhouse-Geisser correction was used when Mauchly's test indicated that the assumption of sphericity had been violated. The Bonferroni correction was applied to control for multiple comparisons.

2.2 Emotional Measures

Results of the two-way repeated measures ANOVA for valence ratings indicated a significant main effect of the type of auditory excerpt, $F(2, 94) = 98.21$, $p < .001$. Contrasts revealed that participants rated music ($p < .001$) and nature sound ($p < .001$) significantly more pleasant than noise. The difference in valence ratings between music and nature sound was non-significant ($p = .656$). There was no significant main effect of pharmacological manipulations on valence ratings, $F(2, 94) = .13$, $p = .883$, and there was no significant interaction between the type of auditory excerpt and pharmacological manipulations, $F(3.37, 158.22) = .61$, $p = .627$, using the Greenhouse-Geisser correction for the interaction. Mean scores are presented in Supplementary Table 4.

Results of the two-way repeated measures ANOVA for liking ratings indicated a significant main effect of the type of auditory excerpt, $F(2, 94) = 111.81$, $p < .001$. Contrasts revealed that participants liked music ($p < .001$) and nature sound ($p < .001$) significantly more than noise. The difference in liking ratings between music and nature sound was non-significant ($p = .579$). There was no significant main effect of pharmacological manipulations on liking ratings, $F(2, 94) = .28$, $p = .753$, and there was no significant interaction between the type of auditory excerpt and pharmacological manipulations, $F(4, 188) = .50$, $p = .735$. Mean scores are presented in Supplementary Table 4.

Results of the two-way repeated measures ANOVA for arousal ratings indicated a significant main effect of the type of auditory excerpt, $F(2, 94) = 17.83$, $p < .001$. Contrasts revealed that participants rated music ($p = .011$) and nature sound ($p < .001$) to be significantly more relaxing (low arousal) than noise. In addition, participants rated nature sound to be significantly more relaxing (low arousal) than music ($p = .020$). There was no significant main effect of pharmacological manipulations on arousal ratings, $F(1.75, 82.37) = .66$, $p = .500$, and there was no significant interaction between the type of auditory excerpt and pharmacological manipulations, $F(4, 188) = .74$, $p = .567$, using the Greenhouse-Geisser correction for the effect of pharmacological manipulations. Mean scores are presented in Supplementary Table 4.

Results of the Pearson correlation analyses for valence, liking, and arousal ratings in relation to pain intensity and pain unpleasantness during noise, nature sound, and music indicated significant correlations between valence and pain unpleasantness scores for noise, liking, and pain unpleasantness scores for noise, and liking and pain unpleasantness scores for music. Additional correlations were non-significant as presented in Supplementary Table 5.

3 Supplementary Figures

Supplementary figures include:

- Supplementary Figure 1: Randomization design (Latin and Graeco-Latin squares)
- Supplementary Figure 2: Paradigm for administration of auditory excerpts and thermal stimuli
- Supplementary Figure 3: Pain intensity and unpleasantness across conditions
- Supplementary Figure 4: Expected pain intensity and unpleasantness across conditions
- Supplementary Figure 5: Associations between perceived and expected pain in the first test condition

Square		1			2		
		Group					
Test day	Period	1	3	5	2	4	6
1	1	Music	Nature	Noise	Music	Nature	Noise
1	2	Nature	Noise	Music	Noise	Music	Nature
1	3	Noise	Music	Nature	Nature	Noise	Music
2	1	Nature	Noise	Music	Noise	Music	Nature
2	2	Noise	Music	Nature	Nature	Noise	Music
2	3	Music	Nature	Noise	Music	Nature	Noise
3	1	Noise	Music	Nature	Nature	Noise	Music
3	2	Music	Nature	Noise	Music	Nature	Noise
3	3	Nature	Noise	Music	Noise	Music	Nature

Pharmacological manipulations
- Haloperidol
- Naltrexone
- Inactive

Supplementary Figure 1. Randomization design (Latin and Graeco-Latin squares)
Participants were randomized by draw into 6 groups of 8 participants allowing for a counterbalanced distribution of experimental conditions: test day (1; 2; 3), periods on each test days (1; 2; 3), auditory excerpts (music, nature sound, noise) and pharmacological manipulations (inactive agent, naltrexone, haloperidol). The box specifying the pharmacological manipulations was blinded until completion of the data analysis.

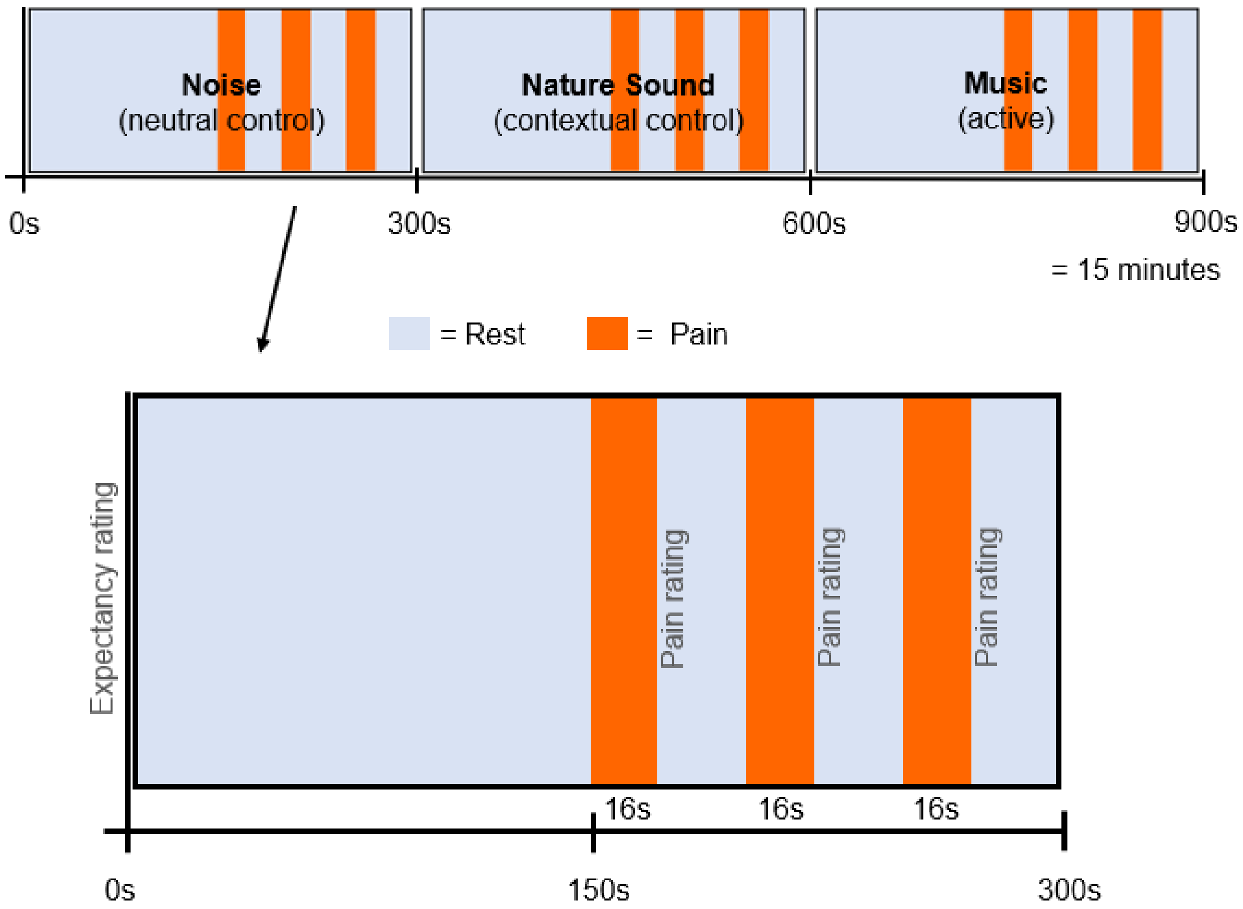

Supplementary Figure 2. Paradigm for administration of auditory excerpts and thermal stimuli During the first 150 s, the participants listened to the auditory excerpt without thermal stimulation. The last 150 s comprised the 3 thermal stimuli with intervening rest intervals. Participants rated their expectations for pain intensity and pain unpleasantness before administering each auditory excerpt and before pain stimuli 1 and 3. Participants rated their perceived pain intensity and pain unpleasantness after each pain stimulus.

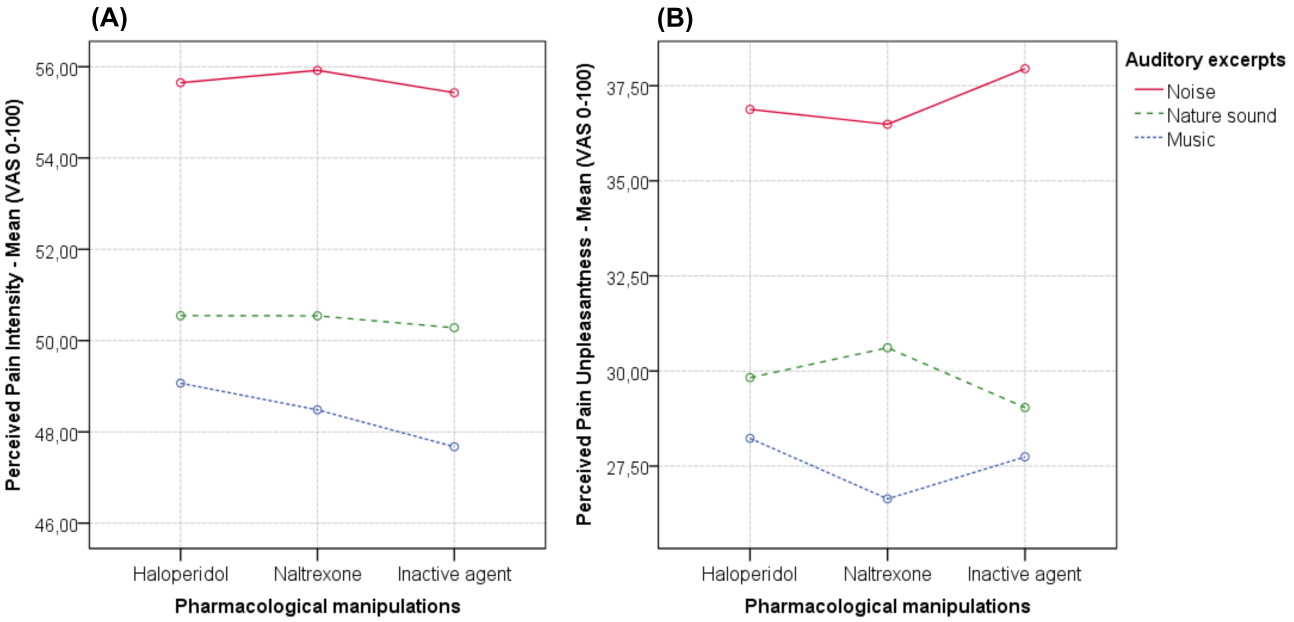

Supplementary Figure 3. Pain intensity and unpleasantness across conditions
Comparisons of noise, nature sound, and music across pharmacological manipulations (regardless the day of administration) on (A) pain intensity and (B) pain unpleasantness indicating a significant main effect of auditory excerpts, a non-significant main effect of pharmacological manipulations and a non-significant interaction.

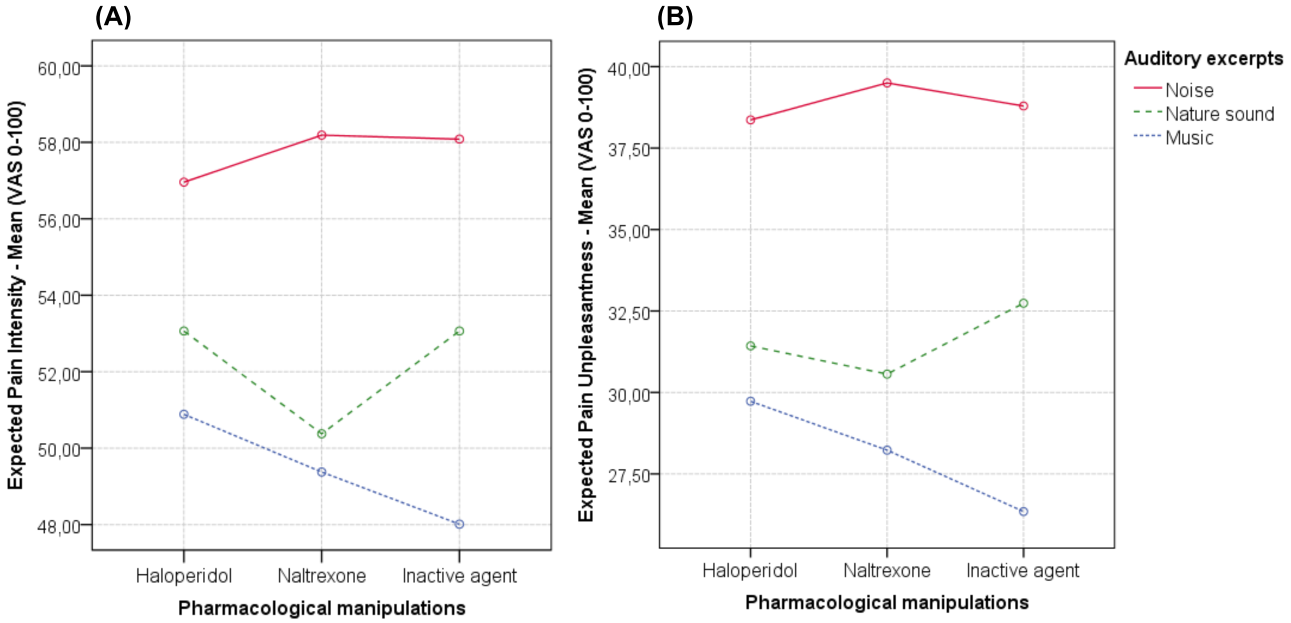

Supplementary Figure 4. Expected pain intensity and unpleasantness across conditions
Comparisons of noise, nature sound, and music across pharmacological manipulations (regardless the day of administration) on (A) expected pain intensity and (B) expected pain unpleasantness indicating a significant main effect of auditory excerpts, a non-significant main effect of pharmacological manipulations and a non-significant interaction.

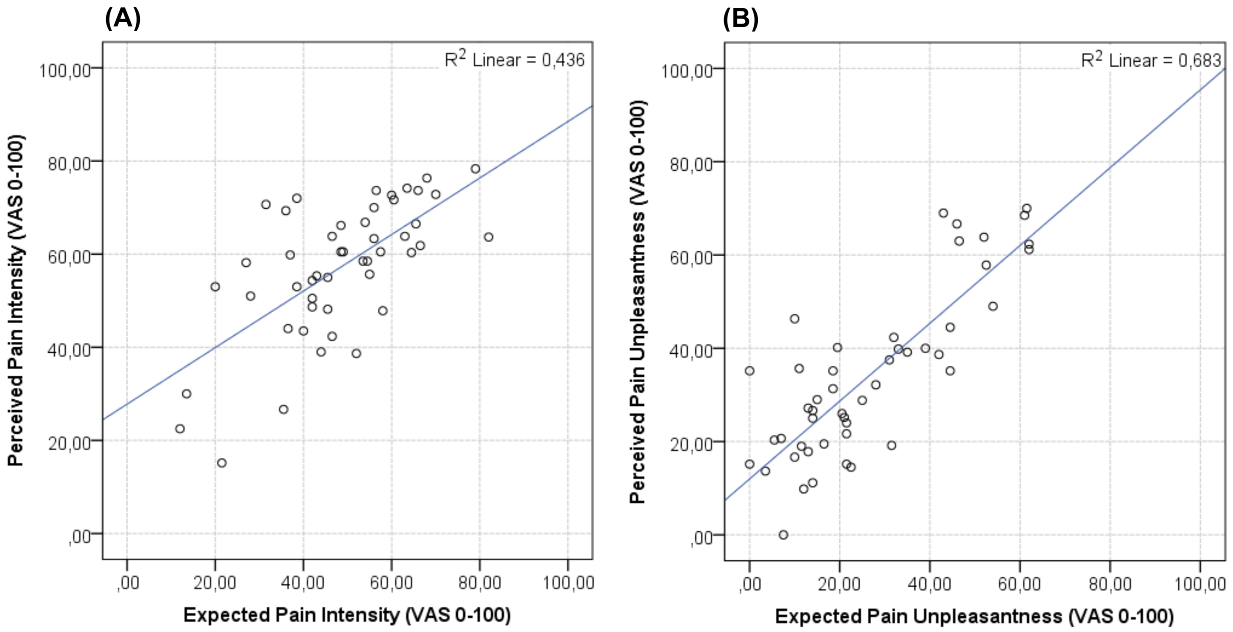

Supplementary Figure 5. Associations between perceived and expected pain in the first test condition

Correlations between (A) expected pain intensity and perceived pain intensity and (B) expected pain unpleasantness and perceived pain unpleasantness in relation to the first auditory excerpt on test day 1.

4 Supplementary Tables

Supplementary tables include:

- Supplementary Table 1: Reports of adverse events (count)
- Supplementary Table 2: Mean scores for perceived pain intensity and pain unpleasantness across auditory excerpts and pharmacological manipulations
- Supplementary Table 3: Mean scores for expected pain intensity and pain unpleasantness across auditory excerpts and pharmacological manipulations
- Supplementary Table 4: Mean scores for valence, liking, and arousal across auditory excerpts and pharmacological manipulations
- Supplementary Table 5: Correlations between valence, liking, and arousal scores and perceived pain intensity and unpleasantness across auditory excerpts

Supplementary Table 1. Reports of adverse events (count)

Symptom	Inactive agent	Naltrexone	Haloperidol
**Drowsiness**	**1**	**5**	**3**
**Dizziness or/and sweating**		**6**	
**Headache**	**1**	**1**	
**Restlessness/concentration difficulties**		**2**	**2**
**Feeling "off"/feeling ill**		**1**	**2**
**Nausea**		**2**	
**Vomiting**		**1***	
**In total**	**2**	**18**	**7**

*To obtain a counterbalanced data analysis and seeing that there was no main effect of the pharmacological manipulations on pain intensity (p = .869) or pain unpleasantness (p = .92), the participant was not eliminated from the data analysis. Naltrexone takes effect after approximately 60 min (7), and the participant vomited after approximately 90 min.

Supplementary Table 2. Mean scores for perceived pain intensity and pain unpleasantness across auditory excerpts and pharmacological manipulations

		Pain Intensity				Pain Unpleasantness			
				95% CI for Mean				95% CI for Mean	
		Mean	SD	Lower	Upper	Mean	SD	Lower	Upper
**Noise**	**H**	55.65	17.60	50.54	60.76	36.88	20.62	30.89	42.87
	**N**	55.92	18.35	50.21	60.99	36.49	21.70	30.18	42.79
	**I**	55.43	18.83	49.96	60.90	37.95	21.73	31.64	44.26
**Nature sound**	**H**	50.55	16.56	45.74	55.35	29.83	17.22	24.83	34.83
	**N**	50.54	17.77	45.20	55.64	30.61	18.87	25.13	36.09
	**I**	50.28	17.95	45.07	55.49	29.04	18.35	23.71	34.37
**Music**	**H**	49.07	17.09	44.10	54.03	28.23	18.64	22.82	33.64
	**N**	48.48	18.25	42.90	53.62	26.64	19.93	20.85	32.43
	**I**	47.67	18.39	42.33	53.01	27.74	18.41	22.40	33.09

Haloperidol: H; Naltrexone: N; Inactive pill: I.

Supplementary Table 3. Mean scores for expected pain intensity and pain unpleasantness across auditory excerpts and pharmacological manipulations

		Pain Intensity				Pain Unpleasantness			
				95% CI for Mean				95% CI for Mean	
		Mean	SD	Lower	Upper	Mean	SD	Lower	Upper
**Noise**	**H**	56.96	16.86	52.06	61.86	38.36	20.75	32.34	44.39
	**N**	58.19	19.05	52.66	63.72	39.50	20.95	33.42	45.58
	**I**	58.08	18.28	52.78	63.39	38.79	20.79	32.75	44.83
**Nature sound**	**H**	53.06	16.29	48.33	57.79	31.43	18.84	25.96	36.90
	**N**	50.38	17.86	45.19	55.56	30.56	20.24	24.69	36.44
	**I**	53.06	17.39	48.01	58.11	32.74	19.06	27.20	38.28
**Music**	**H**	50.89	17.08	45.93	55.84	29.73	18.55	24.34	35.12
	**N**	49.38	19.03	43.85	54.90	28.23	19.37	22.60	33.85
	**I**	48.01	18.07	42.76	53.26	26.34	17.34	21.31	31.38

Mean scores obtained immediately before participants listened to noise, nature sound and music.
Haloperidol: H; Naltrexone: N; Inactive pill: I.

Supplementary Table 4. Mean scores for valence, liking, and arousal across auditory excerpts and pharmacological manipulations

		Valence		Liking		Arousal	
		**Mean**	**SD**	**Mean**	**SD**	**Mean**	**SD**
**Noise**	**H**	3.69	1.99	2.60	2.17	4.58	1.47
	**N**	3.90	2.45	2.54	2.34	4.65	1.56
	**I**	3.75	2.08	2.56	2.21	4.38	1.83
**Nature sound**	**H**	7.71	2.08	7.00	2.54	2.42	1.97
	**N**	7.73	2.07	7.21	1.91	2.54	2.20
	**I**	7.69	1.81	7.04	1.92	2.79	2.18
**Music**	**H**	8.10	1.69	7.67	1.99	3.33	2.31
	**N**	7.94	1.93	7.60	1.76	3.71	2.25
	**I**	8.27	1.18	7.40	1.76	3.48	2.11

Haloperidol: H; Naltrexone: N; Inactive pill: I.

Supplementary Table 5. Correlations between valence, liking, and arousal scores and perceived pain intensity and unpleasantness across auditory excerpts

		PI noise (H,N,I)	PU noise (H,N,I)	PI nature (H,N,I)	PU nature (H,N,I)	PI music (H,N,I)	PU music (H,N,I)
**Valence noise (H,N,I)**	***r***	-.16	-.34*	-.09	-.31*	-.01	-.14
**Valence nature (H,N,I)**	***r***	-.07	-.08	-.19	-.22	-.07	-.04
**Valence music (H,N,I)**	***r***	.01	-.04	.01	-.12	-.12	-.23
**Liking noise (H,N,I)**	***r***	-.21	-.31*	-.08	-.25	-.07	-.15
**Liking nature (H,N,I)**	***r***	-.14	-.09	-.18	-.19	-.11	-.03
**Liking music (H,N,I)**	***r***	-.09	-.12	-.08	-.18	-.23	-.30*
**Arousal noise (H,N,I)**	***r***	.09	.16	-.02	.13	-.04	.05
**Arousal nature (H,N,I)**	***r***	.05	.13	.17	.28	.10	.23
**Arousal music (H,N,I)**	***r***	-.03	-.09	-.07	-.11	.08	.05

Mean pain intensity (PI) and mean pain unpleasantness (PU) scores based on all 3 pain ratings per auditory excerpt and collapsed for all pharmacological manipulations (H,N,I); Haloperidol: H; Naltrexone: N; Inactive pill: I; Nature sound: nature. *$p < .05$

Supplementary References

1. Colloca L, Wang Y, Martinez PE, Christy Chang YP, Ryan KA, Hodgkinson C, et al. OPRM1 rs1799971, COMT rs4680, and FAAH rs324420 Genes Interact with Placebo Procedures to Induce Hypoalgesia. *Pain* (2019) 160:1824-34. doi: 10.1097/j.pain.0000000000001578
2. Villarreal EA, Brattico E, Vase L, Østergaard L, Vuust P. Superior Analgesic Effect of an Active Distraction versus Pleasant Unfamiliar Sounds and Music: The Influence of Emotion and Cognitive Style. *PLoS One* (2012) 7:e29397. doi: 10.1371/journal.pone.0029397
3. Jensen KB, Kaptchuk TJ, Kirsch I, Raicek J, Lindstrom KM, Berna C, et al. Nonconscious Activation of Placebo and Nocebo Pain Responses. *Proc Natl Acad Sci U S A* (2012) 109:15959-64. doi: 10.1073/pnas.1202056109
4. Vuust P, Frith CD. Anticipation is the Key to Understanding Music and the Effects of Music on Emotion. *Behav Brain Sci* (2008) 31:599-600. doi: 10.1017/S0140525X08005542
5. Price DD, Craggs J, Verne GN, Perlstein WM, Robinson ME. Placebo Analgesia is Accompanied by Large Reductions in Pain-Related Brain Activity in Irritable Bowel Syndrome Patients. *Pain* (2007) 127:63-72. doi: 10.1016/j.pain.2006.08.001
6. Peerdeman KJ, van Laarhoven AI, Keij SM, Vase L, Rovers MM, Peters ML, et al. Relieving Patients' Pain with Expectation Interventions: A Meta-Analysis. *Pain* (2016) 157:1179-91. doi: 10.1097/j.pain.0000000000000540
7. Sudakin D. Naltrexone: Not Just for Opioids Anymore. *J Med Toxicol* (2016) 12:71-5. doi: 10.1007/s13181-015-0512-x
